# Fine-Grained Perception for Fundus and Prostate Medical Image Segmentation

**DOI:** 10.3390/s26092879

**Published:** 2026-05-05

**Authors:** Qiao Ba, Jia-Xuan Jiang, Yuee Li, Zhong Wang

**Affiliations:** School of Information Science and Engineering, Lanzhou University, Lanzhou 730000, China; baq2023@lzu.edu.cn (Q.B.); jiangjx2023@lzu.edu.cn (J.-X.J.); liyuee@lzu.edu.cn (Y.L.)

**Keywords:** medical image segmentation, single domain generalization, SAM, fine-grained structural enhancement

## Abstract

Traditional deep learning-based models have achieved promising results in medical image segmentation. However, their performance degrades severely when applied to unseen domains due to variations in imaging protocols, acquisition devices, and patient populations across medical centers, which lead to significant distribution shifts. With the emergence of the Segment Anything Model (SAM), a single model now exhibits significantly improved generalization and adaptability to various image types. Nevertheless, while SAM has learned structure representations from large-scale natural images, it lacks fine-grained structural knowledge specific to the medical imaging domain, remaining relatively invariant across imaging domains. In addition, its structural enhancement is vulnerable to unreliable prompts, and patch-wise inference disrupts structural continuity, leading to suboptimal performance in capturing anatomical details. To address this, we propose a novel Medical Fine-grained Segment Anything Model (termed MedFineSAM), which integrates three key modules: shared fine-grained structural enhancement, which extracts and selectively enhances fine-grained structural features shared between prompts and image embeddings via a structural dictionary; a prompt gating mechanism, which estimates prompt confidence and dynamically adjusts prompt weights to avoid erroneous enhancement; and a structural continuity diffusion in frequency domain (SCFD), which performs frequency-domain smoothing during decoding to alleviate structural discontinuity caused by patch aggregation. Experiments on the fundus benchmark and prostate MRI benchmark demonstrate superior generalization performance, offering new insights into leveraging SAM for single-source domain generalization in medical image segmentation.

## 1. Introduction

Medical image segmentation plays a crucial role in disease diagnosis, clinical assessment, and treatment planning. With the rapid development of deep learning, segmentation models (such as U-Net [1] and nnU-Net [2]) have achieved remarkable performance across multiple medical scenarios. However, the success of deep learning largely relies on the i.i.d. assumption between training and testing samples [3]. In medical image segmentation tasks, this assumption is often violated due to differences in imaging devices and heterogeneity in patient populations [3].

To address the issue of domain shift, domain generalization (DG) methods attempt to learn domain-invariant features from multiple annotated source domains [4,5,6,7], while domain adaptation (DA) methods assume access to unlabeled target domain data to enable feature transfer [8,9,10]. Although DG and DA have demonstrated effectiveness in experimental settings, the former requires expensive multi-source annotations, while the latter presupposes access to target domain data, making both difficult to satisfy in real clinical scenarios. In contrast, single-source domain generalization (SDG) is more realistic: it relies solely on one annotated source domain while enabling cross-center deployment, thereby reducing annotation costs and improving clinical feasibility [11,12,13,14].

Current SDG approaches for medical image segmentation can be broadly divided into two categories. The first type is learning-strategy-based methods, such as contrastive learning [15,16,17] and meta-learning [7,18]. The second type is feature-enhancement-based methods, such as style augmentation [19] and random perturbations [20,21]. Although style augmentation and random perturbations can also be regarded as data-enhancement-based methods, their essential role lies in strengthening the feature extraction ability of the model. While these approaches achieve promising results, they are often designed and trained for specific segmentation tasks. When applied to domains with large distribution gaps or different imaging modalities, their performance significantly degrades [22,23].

Recently, the emergence of Vision Foundation Models (VFMs) has substantially improved both representational capacity and generalization [24,25]. In particular, the Segment Anything Model (SAM) [26,27], trained on over 1 billion masks, demonstrates strong zero-shot generalization and multi-task adaptability in natural images, opening new opportunities for medical SDG segmentation. Several adaptations of SAM to medical imaging have been proposed: DeSAM [28] decouples mask generation from prompt embedding to improve flexibility across prompt types; MedSAM [22] fine-tunes SAM on a large-scale medical image–mask dataset to mitigate distribution gaps between natural and medical images; SAMed [27] employs LoRA-based fine-tuning, freezing the encoder and introducing lightweight bypass modules to adapt SAM [26] to medical tasks. These methods highlight promising directions and show encouraging results, yet they commonly overlook fine-grained structural information that is critical for medical image segmentation, largely because their designs emphasize efficient parameter adaptation and global semantic transfer, while lacking dedicated mechanisms to capture local structural cues. Moreover, the SAM model, although equipped with fine-grained representations learned from natural images, lacks medical fine-grained knowledge and shows a limited ability to capture clinically relevant anatomical details [29,30]. As a result, when directly applied to medical segmentation, its sensitivity to clinically critical details such as lesion boundaries, vessel bifurcations, and subtle anatomical contours remains limited [29,31], particularly in challenging cases such as “lesion boundaries with shadow artifacts” or “blurred organ contours” [32]. In the single-source domain generalization (SDG) task of medical image segmentation, focusing on fine-grained structural information is crucial for improving cross-domain robustness. The reason is that local structures (such as edges, textures, and anatomical contours) are often more stable across different domains than global intensity distributions [33], thus providing reliable cross-domain cues for the model. If only global features are relied upon, models tend to suffer from over-smoothing and boundary blurring in new domains. In contrast, fine-grained modeling can preserve clear lesion boundaries and small structures, thereby significantly enhancing generalization performance [34,35]. Consistent with these findings, facing the task of fundus image segmentation, Meedeniya et al. [36] pointed out in their 2025 systematic review that even state-of-the-art medical segmentation models still fail to achieve satisfactory performance in cross-center fundus optic disc and cup segmentation, mainly due to their inability to capture stable fine-grained structural features across different imaging devices and patient populations. This further motivates us to design a dedicated fine-grained perception module to enhance SAM’s generalization ability in medical scenarios. Facing the task of prostate image segmentation, likewise, a 2024 clinical systematic review from the radiology community further confirmed that this dual challenge of poor cross-domain generalization and insufficient fine-grained structural perception is prevalent across medical imaging modalities: even though 93% of algorithms have achieved expert-level whole-prostate segmentation, the mean Dice score for clinically critical peripheral zone segmentation remains only 0.79, and cross-vendor performance varies drastically due to scanner-specific distribution shifts [37]. Especially, DAPSAM, the SOTA method using SAM [26] in medical segmentation, addresses SAM’s lack of medical fine-grained focus by integrating low-level features into intermediate representations via adapters, which increases sensitivity to details but still lacks the ability to robustly extract domain-invariant fine-grained structures. Directly leveraging the entire low-level feature map often introduces noise, leading to erroneous enhancement.

**Figure 1:**
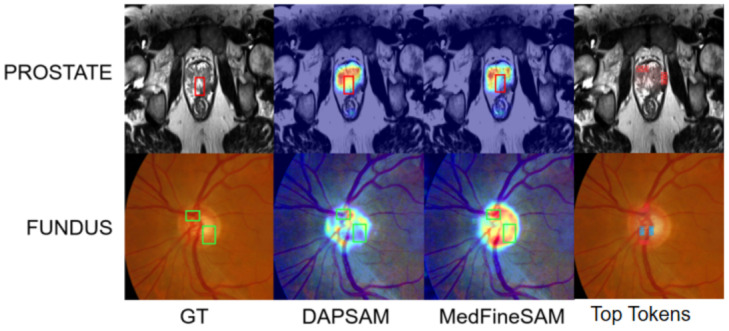
Fundus and prostate CAM heatmaps and top tokens. In fundus images (green-box regions), MedFineSAM is able to focus more effectively on fine-grained structures such as vessels and boundaries, whereas in prostate images (red-box regions), DAPSAM shows stronger responses in relatively smooth-texture areas. This observation indicates that the proposed fine-grained structure enhancement module effectively empowers MedFineSAM to capture richer fine-grained structural features. Meanwhile, the top-token visualization on the far right further shows that, in fundus images, the blue top tokens correspond to the optic disc and the red top tokens correspond to the optic cup, while in prostate images, only the top tokens of the prostate region are displayed. Notably, these high-weight tokens mainly fall within the corresponding organ regions and are concentrated around vessels or segmentation boundaries, further suggesting that the learned tokens possess strong structural representation ability and exhibit a certain degree of robustness.

Therefore, in this paper, we ask: (1) How do we inject domain-invariant medical fine-grained structures into SAM without amplifying noise? (2) How do we suppress unreliable prompt enhancement? (3) How do we repair structural discontinuity introduced by patch-wise inference and fine-grained adjustments? To address these issues, we propose a fine-grained enhancement framework. In the backbone network, fine-grained adapters are embedded into the first two layers to extract structure-sensitive tokens and construct a fine-grained dictionary. The adapters are inserted into the first two layers, as shallow layers encode domain-invariant structural information [17]. The extracted tokens are then used to enhance the fine-grained features of the shallow layers, and the fused representations from these enhanced shallow features are further leveraged in the deeper layers of the backbone, thereby mitigating the loss of fine-grained information, and the resulting attention improvement is illustrated in Figure 1. Moreover, in medical segmentation, the structural enhancement process of SAM is highly sensitive to unreliable or ambiguous prompts—when the prompt features do not align with the true anatomical structures, the model may amplify erroneous local cues through cross-attention, leading to degraded boundary quality. Therefore, in the prompt generation branch, we select the Top-λ proportion of fine-grained tokens from the backbone-constructed fine-grained dictionary that exhibit the highest similarity to the prompt prototype, and use these tokens to reinforce the prototype, thereby aligning prompt features with the true anatomical structures. Nonetheless, since some fine-grained tokens may still carry residual style cues, the enhancement process can be unstable. To address this, we introduce a prompt gating mechanism, which adaptively reweights the contribution of prompts based on their consistency with the image embedding. Furthermore, fine-grained enhancement introduces spatial discontinuities, manifested as fragmented mask boundaries, blocky artifacts in small regions, or jagged edges. Cross-domain style discrepancies (e.g., noise, contrast distribution) further amplify these distortions. To mitigate such effects, we incorporate SCFD, a frequency-domain adaptive module placed before the mask decoder, which performs frequency-domain smoothing to enhance structural continuity and suppress high-frequency noise.

**Contributions.** Our main contributions are threefold: (1) We propose MedFineSAM, a SAM-based fine-grained perception framework for SDG medical image segmentation. (2) We design and integrate three key components, shared fine-grained structural enhancement, prompt gating mechanism, and structural continuity diffusion in frequency domain (SCFD), enabling robust fine-grained alignment and processing. (3) Extensive experiments across multiple tasks and benchmarks demonstrate that MedFineSAM significantly outperforms existing approaches and establishes new state-of-the-art results, validating its clinical potential.

## 2. Methods

### 2.1. Problem Definition and Framework Overview

In this work, we focus on the challenging setting of single-source domain generalization (SDG) for medical image segmentation. Formally, the training set is drawn from a single source domain $D^{s}={\left\{\left(x_{i}^{s},y_{i}^{s}\right)\right\}}_{i=1}^{N_{s}}$, where $x_{i}^{s}$ and $y_{i}^{s}$ denote the source image and its corresponding label. The trained model is then directly evaluated on a collection of unseen target domains $D^{t}=\left\{D_{1}^{t},D_{2}^{t},\ldots ,D_{n}^{t}\right\}$. Unlike conventional domain generalization that leverages multiple labeled source domains, SDG assumes that only a single annotated domain is available for training, making it more consistent with real-world clinical scenarios where collecting diverse multi-center data is constrained by privacy, annotation cost, and scanner heterogeneity.

We propose a fine-grained structural perception framework that improves the cross-domain segmentation performance of SAM under single-source training. The overall workflow is as follows:

In the image encoding branch, low-level features often fail to capture cross-domain stable fine-grained structures. To address this, we design a learnable fine-grained structural dictionary that serves as a cross-domain prior. Concretely, the dictionary is factorized into low-rank form and injected into the first two frozen encoder layers, where tokens are selected by similarity for feature compensation and then fused with the original features in a residual manner. The enhanced features from these two layers are subsequently fused and propagated to the following layers, enabling deeper representations to be enriched by fine-grained structural cues.

In the prompt branch, conventional prompt vectors lack sensitivity to cross-domain fine-grained structures. We therefore leverage the shared dictionary to guide prompt generation. Specifically, an instance-level prototype is obtained through global pooling, matched against the dictionary, and the Top-λ tokens are aggregated through an MLP to produce a fine-grained prototype. This prototype is then used to generate a similarity-based activation map, which is compressed into a dynamic prompt vector to spatially guide the decoder.

During the fusion of prompt and image embeddings, we introduce a prompt gating mechanism to adaptively balance their contributions. Since prompt features and image features may not always be equally reliable, the gating weights dynamically adjust their influence, effectively suppressing noise and enhancing alignment.

Finally, in the decoder branch, fine-grained compensation may introduce boundary discontinuities or cross-domain inconsistencies. To mitigate this, we propose a structural continuity diffusion in frequency domain (SCFD) module. By performing smoothing in the frequency domain, SCFD alleviates local discontinuities while preserving cross-domain alignment and structural consistency.

**Figure 2:**
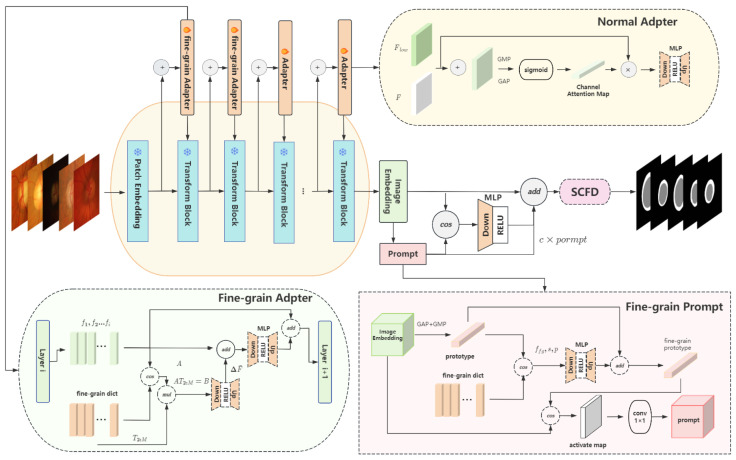
The proposed MedFineSAM pipeline fine-tunes SAM through a generalization framework. It integrates fine-grained adapters (**bottom left**), normal adapters (**top right**) for robust features, a prompt generation module (**bottom right**) for fine-grained source-domain prototypes in target segmentation, and an SCFD module (far right) to smooth results (detailed structure in subsequent sections).

Overall, our framework leverages the synergy of fine-grained structural enhancement, a prompt gating mechanism, and a structural continuity diffusion in frequency domain (SCFD) module, achieving superior cross-domain segmentation performance on nine retinal fundus datasets and six prostate MRI datasets. An overview of the proposed MedFineSAM pipeline is illustrated in Figure 2.

### 2.2. Fine-Grained Structural Enhancement: Instance-Level Structure Awareness

Although SAM has demonstrated the ability to capture fine-grained structures in natural images thanks to its large-scale pretraining, it lacks explicit knowledge of medical fine-grained anatomy. By explicitly enhancing the capture of such fine-grained anatomy structures and aligning them with SAM’s high-level semantic representations, it is possible to significantly improve cross-domain robustness and ensure sharper boundary delineation in the SDG setting.

Motivated by these observations, inspired by Low-Rank Adaptation (LoRA) [38], we propose a fine-grained structural enhancement mechanism. Instead of directly re-parameterizing all channels or relying on global class prototypes, we introduce a set of low-rank, learnable tokens *T*, each corresponding to a unique anatomical pattern or morphological variation. Encoder features interact with these tokens through dot-product similarity and weighted aggregation, generating a fine-grained structural compensation at each spatial location. This design enables efficient adaptation to instance-level structural variations with only a small number of additional parameters.

For the fine-grained features learned in the first two backbone layers, we construct a shared fine-grained dictionary, which is injected into both the image encoder adapters and the prompt branch. This shared module learns fine-grained image structures and maintains a fine-grained structural dictionary that stores anatomical prototypes and morphological variations, supporting both inter-branch and inter-sample sharing. During inference, the model retrieves structurally similar tokens from the dictionary, enabling alignment of the image encoder and prompt encoder under the same structural priors and enhancement strategies.

At the beginning of training, a set of learnable tokens is initialized for each layer:

(1) $$T_{i}=\left\{t_{1},\ldots ,t_{M}\right\}\in R^{M\times C}.$$

To reduce parameters and redundancy, while highlighting cross-modal stable structures, each token matrix is factorized at a low rank:

(2) $$T_{i}=A_{i}B_{i},\;A_{i}\in R^{M\times r},\;B_{i}\in R^{r\times C},\;r\ll C.$$

Tokens are initialized from a zero-mean Gaussian distribution (σ=0.01), ensuring negligible initial compensation. Despite being trained solely on a single source domain, the dictionary learns domain-invariant structures through two design choices: (i) adapters are restricted to the first two shallow layers, where features encode low-level geometric cues rather than domain-specific styles; (ii) the silent token mechanism allows ambiguous patches to bypass enhancement, preventing the dictionary from absorbing spurious style correlations. The low-rank factorization $T_{i}=A_{i}B_{i}$ further acts as an implicit regularizer, guiding tokens toward the principal structural modes of the source domain.

#### 2.2.1. Fine-Grained Structural Adaptation (Image Branch)

The backbone network remains frozen; only $A_{i}$, $B_{i}$, and a small MLP are updated by supervised loss. Thus, the token matrix becomes the sole carrier of structural priors, progressively absorbing stable cross-modal geometric prototypes [7,17].

After training, $T\in R^{M\times C}$ constitutes the fine-grained structural dictionary. The first row is a silent token (a global “no-operation” token) that does not participate in local enhancement; rows 2–M are structural tokens used for local compensation. Given encoder features $F\in R^{N\times C}$ (*N* flattened spatial positions, *C* channels), residual structural enhancement is applied:

(3) $$F^{\prime }=F+\Delta F$$

where ΔF=R(F) is generated by retrieval and aggregation from the dictionary, computed as follows:

##### Structural Correlation

Dot-product similarity with Softmax normalization:

(4) $$A=\mathrm{softmax}\;\left(\frac{FT^{⊤}}{\sqrt{C}}\right)$$

yielding weights $A\in R^{N\times M}$.

##### Local Structural Aggregation

Weights are applied only to local tokens (rows 2–M), generating a compensation descriptor:

(5) $$B=A\;T_{2:M}.$$

The probabilities associated with the silent token A[:,1] are excluded, preventing erroneous enhancement when no suitable match exists in the dictionary.

##### Nonlinear Adjustment

*B* is fed into a shared two-layer MLP (with activation functions) [39] to obtain ΔF. Regions highly correlated with source-domain structures receive stronger compensation, while uncertain regions are enhanced less.

(1) First transformation (local compensation draft)
  (6) $$\Delta \bar{F}=BW_{T}+b_{T},$$
  where $W_{T}\in R^{C\times C}$, $b_{T}\in R^{C}$ are learnable parameters.
(2) Second transformation (combined with original features)
  (7) $$\Delta F=\left(\Delta \bar{F}+F\right)W_{f}+b_{f},$$
  where $W_{f}\in R^{C\times C}$, $b_{f}\in R^{C}$.

However, as layers go deeper, features become increasingly semantic and are less capable of directly capturing fine low-level structures. Therefore, after the first two layers we revise the fine-grained adapter strategy: the compensations obtained by the shallow fine-grained structural enhancement module, $\Delta F^{\left(1\right)}$ and $\Delta F^{\left(2\right)}$, already encode *cross-domain stable, instance-level* structure. Starting from the 3rd layer, we *reuse* this shallow compensation as the “low-level feature” injected into deeper layers, and adopt DAPSAM’s [40] *channel filtering + lightweight adapter* pipeline to complete the updates. Unlike DAPSAM [40], we *do not* use the Flow projected from the initial embedding $e_{0}$; instead, we inject the instance-level structural compensation derived via fine-grained retrieval and non-linear adjustment, avoiding repeated injection of domain-related style/noise.

##### Aggregation and Projection of Shallow Compensation


(8) $${\Delta \bar{F}}_{\mathrm{low}}\;=\;\mathrm{Proj}\;\left(\mathrm{Agg}\left[\Delta F^{\left(1\right)},\;\Delta F^{\left(2\right)}\right]\right)$$

We adopt a lightweight learnable scalar weighting that adds negligible overhead while adapting across data distributions to favor the more reliable layer:

(9) $${\mathrm{Agg}}_{\alpha }\left[\Delta F^{\left(1\right)},\Delta F^{\left(2\right)}\right]=\alpha \;\Delta F^{\left(1\right)}+\left(1-\alpha \right)\;\Delta F^{\left(2\right)},\;\alpha \in \left[0,1\right].$$

The shallow compensations $\Delta F^{\left(1\right)},\Delta F^{\left(2\right)}\in R^{N\times C}$ are first aggregated by Agg, then projected by $\mathrm{Proj}\left(X\right)=XW_{p}+b_{p}$ with $W_{p}\in R^{C\times C},\;b_{p}\in R^{C}$ for statistical alignment, yielding a reusable *structural shortcut* ${\Delta \bar{F}}_{\mathrm{low}}\in R^{N\times C}$. This vector condenses denoised, instance-level structural cues, which are more target-aligned and cross-domain stable than directly using $e_{0}\;\to \;$ Flow.

##### Fusion with Current-Layer Features


(10) $$F_{\mathrm{fuse}}^{\left(l\right)}\;=\;F^{\left(l\right)}\;+\;{\Delta \bar{F}}_{\mathrm{low}},\;l\geq 3$$

At layer l≥3, the structural shortcut is injected into the intermediate representation $F^{\left(l\right)}\in R^{N\times C}$ via a residual fusion, producing $F_{\mathrm{fuse}}^{\left(l\right)}$. This preserves semantic content while complementing deep features with shallow structural cues that are otherwise hard to recover.

##### Channel-Attention Filtering

After fusing the structural shortcut $\Delta F_{\mathrm{low}}$ with the current layer features $F^{\left(l\right)}$, we apply channel-attention filtering to adaptively emphasize structure-relevant channels while suppressing redundant or noisy ones. This is particularly important in cross-domain scenarios where certain channels may encode domain-specific artifacts rather than domain-invariant structural information.

The channel weights are computed by aggregating both global average and max pooling responses:

(11) $$w^{\left(l\right)}=\sigma (\mathrm{GAP}\left(F_{\mathrm{fuse}}^{\left(l\right)}\right)+\mathrm{GMP}\left(F_{\mathrm{fuse}}^{\left(l\right)}\right)).$$

The filtered features are then obtained via channel-wise multiplication:

(12) $$F_{\mathrm{filtered}}^{\left(l\right)}=w^{\left(l\right)}⊙F_{\mathrm{fuse}}^{\left(l\right)}$$

where ⊙ denotes element-wise multiplication with broadcasting along spatial dimensions. This operation effectively rescales each channel proportionally to its estimated relevance for structural representation, enhancing the model’s focus on domain-invariant anatomical patterns before the subsequent lightweight adapter processing.

##### Residual Update via Lightweight Adapter


(13) $$F^{\prime (l)}\;=\;F_{\mathrm{filtered}}^{\left(l\right)}\;+\;\mathrm{MLP}\;\left(F_{\mathrm{filtered}}^{\left(l\right)}\right)$$

A two-layer bottleneck MLP (Linear–Act–Linear) performs the lightweight adaptation and residual update, balancing efficiency and nonlinearity to effectively absorb structural compensation.

##### Summary of Advantages

(i) Learning domain-invariant structures from a single source: tokens are initialized from a zero-mean Gaussian distribution (σ=0.01) and factorized in low-rank to capture principal structural modes. By confining the dictionary to shallow layers that encode low-level geometric cues, the tokens naturally converge toward domain-agnostic anatomical primitives. The silent-token mechanism further ensures that ambiguous patches bypass enhancement, preventing the dictionary from absorbing spurious style correlations. (ii) Efficient enhancement: heavy computation (retrieval and nonlinearity) is confined to shallow layers, while deeper layers reuse ${\Delta \bar{F}}_{\mathrm{low}}$ and a lightweight adapter. (iii) Stronger cross-domain stability and generalization: the injected signal is an instance-level structural shortcut rather than a static $e_{0}$-projected Flow, leading to more robust adaptation under distribution shifts.

#### 2.2.2. Fine-Grained Structural Alignment (Prompt Branch)

On the prompt side, we first obtain an instance-level prototype *p* from the initial prompt embedding *P* through GAP or GMP. This prototype is matched with the structural dictionary *T* through cosine similarity, followed by Softmax normalization to obtain the weight vector:

(14) $$\alpha =\mathrm{softmax}\;\left(\frac{pT^{⊤}}{\sqrt{C}}\right),\;\alpha \in R^{1\times M}.$$

It should be noted that the tokens in the dictionary may contain not only structural information but also style or noise components. Theoretically, domain-invariant anatomical structures exhibit higher intra-class similarity and lower intra-sample variance compared to domain-specific style artifacts and noise. Therefore, tokens with higher similarity to the prompt prototype are statistically more likely to encode stable structural features, while low-similarity tokens predominantly correspond to domain-specific noise or irrelevant background patterns. To avoid such interference, we introduce a ranking-based filtering mechanism on the similarity distribution α: tokens are first sorted according to their similarity scores, and only the top λ proportion of the most similar tokens are preserved (λ∈(0,1] is a learnable parameter). In this way, the model preferentially leverages tokens that are more similar to the prototype, which can generally be regarded as domain-invariant structural features, thereby making the enhancement more targeted:

(15) $$f_{fg}=\mathrm{Top-}\lambda \left(\alpha ,T_{2:M}\right)$$

where Top-λ(·) denotes the operation of retaining only the top λ proportion of tokens with the highest similarity scores for aggregation.

Next, the weight distribution α can also be used to define the prompt confidence *s*, which measures the consistency between the current prototype and the structural priors. By concatenating the three components $\left[p,f_{fg},s\right]$ and passing them through a lightweight residual MLP, we obtain the fine-grained prompt prototype:

(16) $$p^{fg}=p+W\;\left([p,f_{fg},s]\right),$$

where *W* denotes a nonlinear MLP.

Subsequently, the cosine similarity between $p^{fg}$ and the image embedding *F* is computed to obtain the activation map, $A^{prompt}=\cos\left(p^{fg},F\right)$, which highlights the regions requiring focused attention. Finally, the activation map is concatenated with the original image embedding and the fine-grained prototype $f_{fg}$ along the channel dimension, and fused with a 1×1 convolution to generate the final prompt embedding.

Based on the pooled single-token prompt, the fine-grained enhancement mechanism not only provides global guidance but also injects structural priors, thereby compensating for the backbone’s tendency to produce overly smoothed representations due to its reliance on local patches. Compared with directly using image embeddings, this method exhibits stronger structural selectivity, imposing global constraints on critical anatomical regions and preventing the model from deviating from plausible anatomical patterns. Meanwhile, through the “similarity ranking + Top-λ” mechanism, the network can automatically ignore style and noise tokens inconsistent with the prototype and utilize only highly similar structural tokens for compensation, thereby further improving the stability and generalization ability of cross-domain segmentation.

Through this token generation and structural-prior absorption process, the fine-grained module achieves instance-level structural awareness and cross-domain fine-grained structural sharing, thereby enhancing the recognition and representation of anatomical details (as shown in Figure 1). On one hand, each image patch receives specific enhancements relevant to its fine structures, compensating for the “one-size-fits-all” limitation of standard adapters. On the other hand, when certain organs or tissues in the target domain become less salient or undergo morphological changes (i.e., structures unseen in the source domain), the module provides reasonable compensation using learned structural priors; when differences are too large, a silent token [41] is activated to prevent harmful over-enhancement. Importantly, the fine-grained module introduces only lightweight parameters (low-rank token matrices and small MLPs), thereby significantly improving cross-domain performance while maintaining minimal complexity.

It is worth noting that our method is inspired by the ReINS strategy [41], which enhances features through interactions between learnable structural tokens and encoder representations. Unlike ReINS, which applies tokens broadly across all layers, we consider that SAM and its fine-tuned variants already possess strong global style modeling capabilities. Therefore, we introduce structural tokens only in the shallow layers (1st and 2nd) to construct the fine-grained dictionary. Furthermore, in the prompt branch, we integrate the Top-λ mechanism to achieve coordinated enhancement between backbone features and the prompt-constructed feature vectors. This modification keeps the framework lightweight while effectively compensating for SAM’s limitations in fine-grained structural modeling.

### 2.3. Prompt Gating Mechanism: Fine-Grained Prompt Alignment

In addition to employing the Top-λ strategy to enhance structural tokens from the dictionary, we introduce a secondary safeguard mechanism to prevent semantic deviations caused by erroneous prompts. The Top-λ filtering operates in the token-level feature space and only captures local structural resemblance, but it cannot guarantee global semantic consistency between the enhanced prompt and the entire image. In SAM’s cross-attention mechanism, even a small portion of mismatched prompt features can be amplified and propagated to the entire segmentation mask, leading to catastrophic degradation in boundary quality. As a result, even if the selected tokens are locally reasonable, the entire prompt may still deviate globally in the image embedding space, since local similarity does not necessarily ensure global semantic consistency; for example, it may activate spurious structures or irrelevant background regions. To address this issue, we propose a prompt gating mechanism.

Let $P\in R^{C\times H\times W}$ denote the generated prompt features, which have the same dimensionality as the image encoder embeddings $X\in R^{C\times H\times W}$. At each spatial location (i,j), we compute their consistency via cosine similarity:

(17) $$S_{i,j}=\frac{\langle P_{:,i,j},\;X_{:,i,j}\rangle }{∥P_{:,i,j}∥\;∥X_{:,i,j}∥}.$$

We then apply average pooling to *S* to obtain a global matching score:

(18) $$a=\frac{1}{H\;W}\sum_{i,j}S_{i,j}.$$

Finally, we concatenate the global average representation of the prompt features $\bar{P}\in R^{C}$ with the matching score *a* to form $h=\left[\bar{P};a\right]\in R^{C+1}$. This vector is fed into a one-layer MLP with ReLU and Sigmoid activations to yield the confidence score c∈[0,1]:

(19) $$c=\sigma \;\left(\mathrm{ReLU}(W_{1}\;h+b_{1})\right).$$

A higher *c* indicates stronger consistency between the generated prompt and the current image, thus assigning higher trust to the prompt; conversely, a lower *c* reduces the influence of the prompt.

Prompt gating serves as a global semantic consistency regulator between the prompt and image embeddings. It evaluates the alignment between the entire prompt representation and the image embedding distribution, and adaptively down-weights unreliable prompts whose structural or semantic patterns are inconsistent with the image features. This effectively suppresses erroneous global enhancement and mitigates its negative impact on segmentation performance. The gating mechanism employs purely soft weighting via linear interpolation (27) without any hard threshold for prompt rejection, allowing unreliable prompts to contribute proportionally less rather than being abruptly discarded.

### 2.4. Structural Continuity Diffusion in Frequency Domain (SCFD)

After the preceding structural enhancement and confidence-guided prompt–image fusion, the model has fine-grained perception and reinforcement of key anatomical structures, along with robustness to local or global domain shifts. However, because structural enhancement applies spatially differentiated adjustments, features lose spatial continuity, manifesting as discontinuous mask boundaries, blocky seams in small regions, and even jagged artifacts. Meanwhile, cross-domain style differences (e.g., noise and contrast distributions) can further amplify such discontinuities and perturbations.

To address these issues, we introduce the structural continuity diffusion in frequency domain (SCFD) module, which performs adaptive smoothing in the frequency domain to effectively connect and fuse the fine structures produced by earlier stages, enabling smooth stitching of both global and local structures, and improving boundary continuity and cross-domain consistency [42].

#### 2.4.1. PDE-Based Frequency Denoising

The foundation of SCFD is the analytical formulation of the heat conduction equation, whose frequency-domain solution was rigorously established in the *Heat* paper [42]. Specifically, when heat diffusion with diffusion time *t* is applied to an initial feature map U(0); each frequency component is attenuated according to

(20) $$\exp[-\kappa \left(\omega_{x}^{2}+\omega_{y}^{2}\right)t],$$

where $\left(\omega_{x},\omega_{y}\right)$ denote the frequency coordinates and κ is the diffusion coefficient. High-frequency components (larger ω) decay rapidly toward zero, while low-frequency components (smaller ω) are better preserved, approaching one.

The analytical form of Equation (20) has been rigorously derived in [42]; therefore, we directly adopt this proven frequency-domain diffusion formulation without re-deriving it. This exponential decay term represents the analytical solution of the two-dimensional heat diffusion PDE in the frequency domain. Moreover, ref. [42] discretizes this continuous solution by employing the 2D discrete cosine transform (DCT) and its inverse (IDCT) to approximate the Fourier transform pair under Neumann boundary conditions, ensuring that spatial information near image borders is preserved.

We follow this discrete formulation and implement SCFD using the fast DCT-II/IDCT pair. By setting the diffusion time step Δt=1 and absorbing κ into the exponential kernel, a single SCFD operation corresponds to one-step diffusion (t=1). On top of this physically grounded model, we further extend the diffusion coefficient κ into a learnable, content-adaptive tensor *K*, enabling adaptive smoothing across frequencies and channels for visual representations.

#### 2.4.2. Structure-Aware Temperature Modulated Frequency Embedding (STM-FE)

To achieve frequency-domain smoothing that is both cross-domain consistent and structurally continuous, we propose the Structure-aware Temperature Modulated Frequency Embedding (STM-FE) module, which extends the standard heat-diffusion model with temperature adjustment and boundary-awareness mechanisms. STM-FE serves as the parameterization head of SCFD: it produces the adaptive diffusion coefficients *K* and temperature τ, which control the frequency-domain heat diffusion applied in Equation (28). Note that the feature map src used in STM-FE is defined later in Equation (27) in the “Decoder Integration with Confidence-Guided Fusion” section, where it represents a confidence-weighted fusion of prompt_emb and image_emb.

(1) Frequency embedding via frequency value embeddings (FVEs): Instead of predicting the frequency-domain attenuation map from a globally pooled prototype vector, we follow the frequency-domain design in [42] and introduce learnable *Frequency Value Embeddings* (FVEs) directly defined on the spectrum. Concretely, for a spectrum of size $H_{f}\times W_{f}$, STM-FE maintains a learnable tensor

(21) $$\mathrm{FVE}\in R^{H_{f}\times W_{f}\times D},$$

where each location (x,y) is associated with an embedding vector $e_{x,y}\in R^{D}$ that encodes prior smoothing strength for that frequency. A shared linear mapping Φ(·) projects FVEs to a scalar base diffusion coefficient at each frequency location, yielding a base tensor $K^{\mathrm{base}}\in R^{H_{f}\times W_{f}}$:

(22) $$K^{\mathrm{base}}\left(x,y\right)=\Phi \left(e_{x,y}\right).$$

This frequency-wise prior is shared across samples and broadcast along the batch and channel dimensions, providing a physically meaningful initialization for diffusion strengths over the spectrum.

(2) Stabilized coefficient tensor with boundary gating: Directly using $K^{\mathrm{base}}$ as diffusion coefficient may still cause negative or unstable values. To ensure non-negative and smooth diffusion strengths, we apply a *Softplus* activation and introduce a boundary-aware gate *G* to modulate the smoothing strength near anatomical edges:

(23) $$K=\mathrm{Softplus}\;\left(K^{\mathrm{base}}\right)⊙G,\;G=1-\lambda \;\sigma \;\left(DCT(|\nabla \mathrm{src}|)\right),\;\lambda \;\in \left[0,1\right].$$

Here, Softplus activation guarantees K≥0, while the gate *G* injects image-dependent modulation into the frequency-wise prior, yielding a structure-aware and content-adaptive diffusion tensor.

To compute the spatial gradient magnitude |∇src|, we adopt a standard first-order finite-difference operator commonly used in edge-aware diffusion:

(24) $$\nabla_{x}\mathrm{src}\left(i,j\right)=\mathrm{src}\left(i,j+1\right)-\mathrm{src}\left(i,j\right),\;\nabla_{y}\mathrm{src}\left(i,j\right)=\mathrm{src}\left(i+1,j\right)-\mathrm{src}\left(i,j\right),$$

and the gradient magnitude is obtained as

(25) $$\left|\nabla \mathrm{src}\right|=\sqrt{{\left(\nabla_{x}\mathrm{src}\right)}^{2}+{\left(\nabla_{y}\mathrm{src}\right)}^{2}}.$$

The resulting gate G∈(0,1] suppresses excessive smoothing at boundary-related frequencies, thereby preserving structural continuity while maintaining the benefits of frequency-domain diffusion.

(3) Adaptive attenuation with temperature modulation: During heat-diffusion filtering, we introduce a learnable global temperature coefficient τ>0 to control the overall diffusion strength:

(26) $${\left(\exp[-\left(\omega_{x}^{2}+\omega_{y}^{2}\right)/\tau ]\right)}^{K}=\exp\;\left[-\frac{K}{\tau }\left(\omega_{x}^{2}+\omega_{y}^{2}\right)\right],\;\tau >0.$$

The temperature τ is trained as a learnable parameter (constrained positive via Softplus). When τ>1, global smoothing weakens; when τ<1, smoothing strengthens. Since K≥0 and exponential decay is monotonic, all frequency weights remain in (0,1], ensuring that diffusion only performs smoothing without amplification.

**Figure 3:**
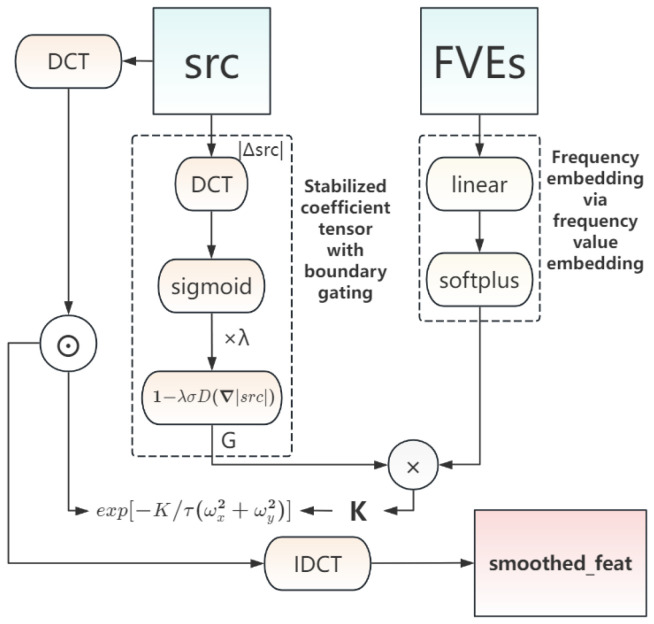
Pipeline of the Structural Continuity Frequency Diffusion (SCFD). The input feature src is processed by the STM-FE module to generate adaptive diffusion coefficients K, which are then used to filter src in the frequency domain via DCT and IDCT, producing the smoothed output smoothed_feat.

Thus, the model assigns larger *K* values (strong attenuation) to high-frequency noise and smaller *K* values (weak attenuation) to low-frequency structures, and regulates mid-frequency details adaptively. The boundary gate *G* reduces over-smoothing around anatomical edges, while the temperature τ provides global control of diffusion strength across samples. This structure-aware, temperature-modulated frequency embedding allows the model to dynamically balance between noise suppression and boundary preservation, achieving smoother and more structurally continuous segmentation results in cross-domain medical image scenarios. The detailed pipeline of the SCFD module is shown in Figure 3.

#### 2.4.3. Decoder Integration with Confidence-Guided Fusion

After frequency-domain filtering, we integrate SCFD into the decoder’s feature-fusion process as follows:

First, use prompt confidence *c* to linearly combine the image encoder features image_emb with the dense prompt features prompt_emb:

(27) src=cprompt_emb+(1−c)image_emb.

When confidence is low, fusion relies primarily on image evidence; when confidence is high, more prompt priors are injected [7,18].

Then apply SCFD to src to obtain smoothed_feat, where the SCFD operation performs frequency-domain diffusion using the adaptive attenuation kernel derived in Equations (21)–(26). Concretely, smoothed_feat is computed by transforming src into the frequency domain via DCT, applying the learned attenuation kernel, and reconstructing the smoothed representation with IDCT:

(28) $$\mathrm{smoothed}\_\mathrm{feat}=\mathrm{IDCT}\;\left(\mathrm{DCT}\left(\mathrm{src}\right)⊙\exp\;\left[-\frac{K}{\tau }\;\left(\omega_{x}^{2}+\omega_{y}^{2}\right)\right]\right),$$

where *K* is the diffusion coefficient tensor defined above, and τ is the global temperature parameter controlling overall smoothing strength. This diffused representation is then added back to the source features in a residual form:

(29) src=src+γsmoothed_feat,

where γ is a learnable gain controlling the contribution of the SCFD-filtered features.

Finally, we fed the SCFD-processed src and the decoder’s prompt tokens into the Transformer decoding layers to produce fine-grained segmentation masks.

**Figure 4:**
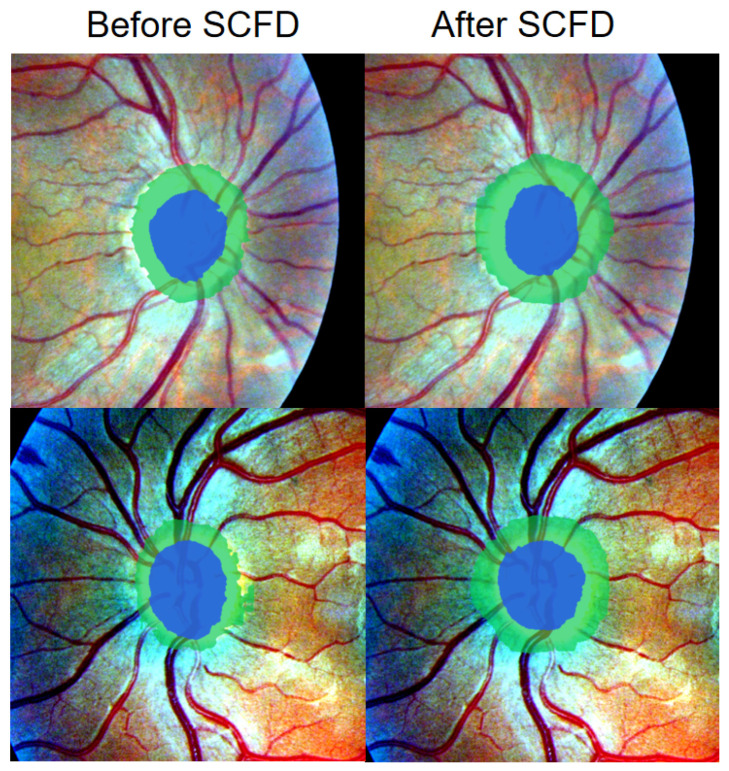
Comparison of segmentation maps before and after using the SCFD method.

From a frequency perspective, SCFD ensures cross-domain consistency and yields smoother, more structurally reliable masks, as reflected in two aspects: Suppressing domain-specific high-frequency noise: the physical heat-diffusion model strongly attenuates high frequencies, removing texture noise and artifacts caused by different scanners or protocols, thereby achieving similar smoothness and continuity across domains and reducing sensitivity to style shifts [19]. The additional temperature coefficient τ provides a global control of smoothing intensity. Preserving key structures and improving boundaries: low-frequency shape and position are largely unaffected, while the adaptive coefficients *K* assign appropriate attenuation to mid-frequency edges, avoiding over-smoothing. The resulting boundaries are more continuous with higher anatomical fidelity, and jagged artifacts are greatly reduced (as shown in Figure 4). Through end-to-end training, its smoothing strength becomes data-adaptive and complementary to the fine-grained structural module: the former regularizes style noise, while the latter enhances structural signals, jointly achieving a clear separation of structure and style.

## 3. Experiments

### 3.1. Dataset Description & Experimental Setting

In this study, we selected two representative cross-domain medical imaging tasks for validation: prostate MRI segmentation and fundus OC/OD segmentation, which are standard benchmarks for generalizable medical image segmentation [16,43,44,45,46].

(1) Prostate MRI dataset

The prostate dataset contains a total of 116 cases collected from six different centers, denoted as: A:(RUNMC); B:(BMC); C:(I2CVB); D:(UCL); E:(BIDMC); and F:(HK). For preprocessing, all slices are resized to a uniform 384 × 384 resolution with consistent voxel spacing. These datasets cover different scanners, imaging protocols, and patient populations, which realistically reflect cross-domain variations and thus provide a solid foundation for studying domain generalization [43]. We adopt the leave-one-out domain generalization strategy: in each experimental run, data from one single center is selected as the exclusive source domain for model training, while data from the remaining five centers are used as target domains for performance validation. This process is repeated sequentially for all six centers, ensuring that each center’s data serves as the source domain exactly once to fully evaluate cross-domain generalization capability across all possible source-target combinations. We adopt the Dice Similarity Coefficient (DSC) as the evaluation metric to quantify segmentation performance across different domains.

(2) Fundus image datasets

For the fundus segmentation experiments, we employed the multi-domain joint dataset RIGA+ [46] and REFUGE [16,44], which consists of seven subsets: BinRushed, Magrabia, MESSIDOR_Base1, MESSIDOR_Base2, MESSIDOR_Base3, REFUGE_Train, and REFUGE_Test. In REFUGE_Train, REFUGE_Test, we employed the same preprocessing method as PCSDG [16]. This forms a complex multi-center, multi-device dataset. In addition, we used two widely recognized standalone datasets:

RIM-ONE_r3 [47]: contains color fundus images of normal, glaucoma-suspect, and glaucoma-confirmed eyes, with expert annotations of the optic disc (OD) and optic cup (OC).

Drishti-GS [45]: includes normal and glaucomatous eyes, and is commonly used for OC/OD segmentation and glaucoma risk assessment.

In our experimental design, we selected RIM-ONE_r3 and Drishti-GS as the source domains for model training, while the remaining eight datasets (including the seven subsets of RIGA+ [46] and REFUGE [16,44] and the unused portions of RIM-ONE_r3/Drishti-GS) were used as target domains for performance evaluation, with DSC as the unified metric. It should be noted that although RIM-ONE_r3 and Drishti-GS are not small in scale, both datasets include a large proportion of normal and glaucomatous eyes, leading to complex case compositions. Furthermore, their imaging devices and acquisition styles differ significantly from the traditional RIGA and REFUGE subsets. As a result, models trained on these two source domains often exhibit poorer generalization when transferred to other target domains. Nevertheless, due to their high clinical relevance and pronounced domain discrepancies, we deliberately selected them as source domains to validate whether the proposed method can enhance cross-domain generalization and segmentation accuracy under the most challenging scenarios.

We first present the quantitative cross-domain segmentation results on the fundus and prostate datasets, respectively. Table 1 summarizes the single-source domain generalization performance on the fundus benchmark, while Table 2 shows the results on the prostate MRI benchmark.

All experiments were conducted based on the Segment Anything Model (SAM) [26] with the ViT-B backbone [53]. During training, the initial learning rate was set to **$5\times 10^{-4}$**, the weight decay coefficient for the AdamW optimizer was set to 0.1, and the adapter level was set to 4. Fine-grained structure enhancement is applied only to the first two encoder layers, where features retain spatial details and geometric structures. Deeper layers are dominated by semantic and style information, and further enhancement there could amplify domain-specific noise [40,41]. Limiting enhancement to early layers thus strengthens structural modeling while preserving robust generalization. We adopted a warm-up strategy following SAMed [27], with warm-up periods set to 250 and 25 for the prostate and fundus datasets, respectively, due to their different training data sizes. Early stopping was applied at 160 epochs with a maximum of 200 epochs. We use DAPSAM [40] as the baseline model.

The training objective follows SAMed [27] and TriD [14], where cross-entropy loss and Dice loss are combined to supervise the entire training process on the source domain:

(30) $$L=\left(1-\beta \right)L_{CE}+\beta L_{Dice}$$

where the hyperparameter β controls the balance between the two terms and is set to 0.8 in our experiments. Other parameters remained consistent with the original SAM configuration.

By training on source-domain datasets and evaluating across multiple target domains, we systematically validate the effectiveness of the proposed method in terms of cross-domain generalization ability.

### 3.2. Ablation Study Results

To quantify the individual contribution of each proposed module and their synergistic effects, we conduct comprehensive ablation studies on both datasets. The ablation results are summarized in Table 3.

#### 3.2.1. Fundus Dataset

**Figure 5:**
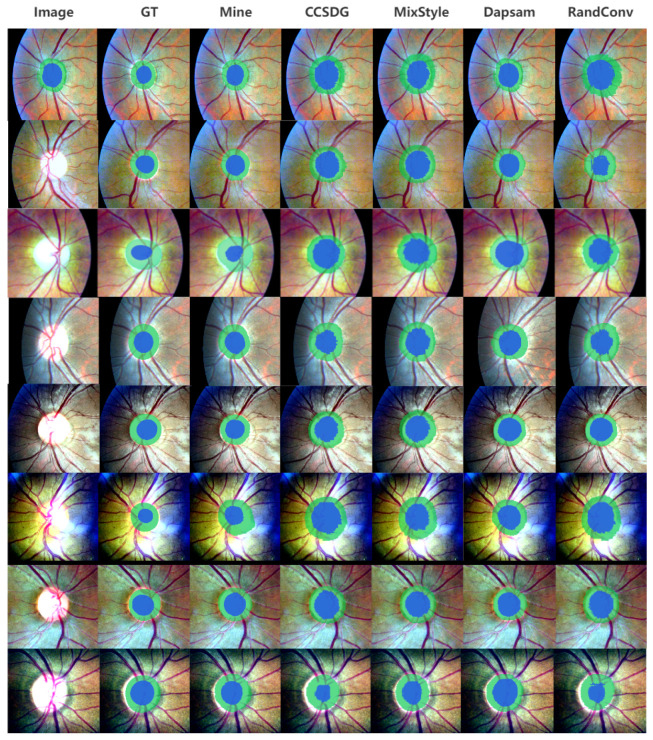
Visual comparison of segmentation results for optic cup-disc across different models, where all selected models are applicable to both optic cup-disc and prostate target domains. In top-to-bottom order, they are sequentially from MESSIDOR_Base1, MESSIDOR_Base2, MESSIDOR_Base3, REFUGE_Train, REFUGE_Test, BinRushed, Magrabia, RIM-ONE_r3.

On the fundus dataset, the average Dice of DAPSAM is 56.06% for Disc and 77.50% for Cup [40]. Results show that single modules yield larger gains compared to the prostate dataset, especially SCFD smoothing (+2.59% for Disc and +0.90% for Cup). Notably, the prompt gating mechanism alone provides substantial improvements on fundus images (+0.95% Disc, +0.59% Cup), considerably larger than its effect on prostate MRI. For glaucoma fundus images, where optic cup/disc boundaries are noisy and blurred, SCFD plays a more important role by reducing high-frequency noise and enhancing boundary continuity. The pronounced benefit of prompt gating in this modality stems from the high variability of fundus images across datasets—differences in illumination, field of view, and pathological changes render generated prompts more susceptible to noise, making adaptive confidence weighting particularly effective. Dual-module combinations are more effective, with fine-grained + SCFD showing the largest improvement (+4.71% for Disc and +1.44% for Cup). When all three modules are enabled, the model achieves the best performance of 61.33% for Disc and 79.16% for Cup, demonstrating the complementarity of the modules. The results of our final model and other competing methods on fundus datasets are visualized in Figure 5.

#### 3.2.2. Prostate Dataset

**Figure 6:**
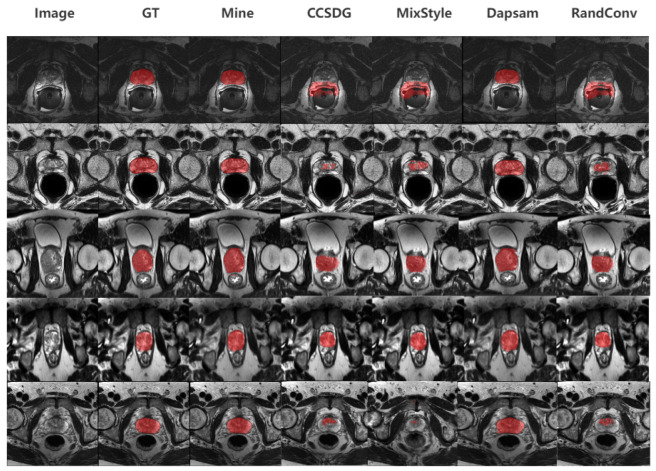
Visual comparison of segmentation results for prostate across different models, where all selected models are applicable to both optic cup-disc and prostate target domains. In top-to-bottom order, they are, sequentially, from BIDMC, BMC, I2CVB, RUNMC, UCL.

On the prostate MRI dataset, the average Dice of DAPSAM is 78.58% [40]. We observe that single modules bring limited improvements. Among them, the fine-grained structure enhancement contributes more clearly (+0.51%), as the prostate contains complex and fine anatomical structures, where fine-grained tokens can better capture local morphology [18]. In contrast, the prompt gating mechanism yields a relatively modest gain on prostate MRI (+0.26%)—substantially smaller than the +0.95% and +0.59% improvements observed for fundus Disc and Cup, respectively. This modality-specific difference arises because prostate anatomy exhibits more consistent global structure across imaging domains; the generated prompts are inherently more reliable, leaving less room for confidence-based weighting to improve upon the baseline. Conversely, fundus images suffer from greater cross-domain variability in illumination, field of view, and pathological appearance, rendering prompts more noise-prone and thus benefiting more from adaptive gating. The combination of two modules further improves performance, with fine-grained + SCFD reaching 79.39% (+0.81%). When all three modules are enabled, the model reaches 79.79%, which is a relative improvement of +1.21% compared with DAPSAM. This indicates that in medical tasks with complex structures and fine boundaries, fine-grained modeling plays a more prominent role. However, its effect is most stable when combined with prompt confidence and SCFD. The results of our final model and other competing methods on prostate datasets are visualized in Figure 6.

While the absolute Dice improvement over DAPSAM appears moderate (+1.21% on prostate and +1.66% to +5.27% on fundus), it is critical to contextualize this gain within the single-source domain generalization (SDG) setting where the baseline performance is already saturated above 78%. Notably, the marginal improvements are not uniformly distributed; as illustrated in Figure 4, the integration of the SCFD module and fine-grained enhancement primarily rectifies structural discontinuities and jagged artifacts—deficiencies that are clinically detrimental yet often poorly penalized by region-based metrics like Dice. Moreover, the ablation study (Table 3) reveals that removing any component leads to a statistically significant degradation in cross-domain consistency (e.g., a drop of 0.81% in prostate when SCFD is omitted, and up to 4.71% in optic disc segmentation). In the context of medical deployment, where false positive boundaries or fragmented masks can mislead diagnostic assessment, the consistent, albeit modest, quantitative gain combined with the qualitative restoration of anatomical continuity substantiates the practical necessity of all three modules.

### 3.3. Model Complexity Analysis

To verify the lightweight nature of the proposed framework as claimed, we provide a detailed quantitative comparison in terms of total parameters, trainable parameters, FLOPs, and inference latency against the standard fully fine-tuned SAM (Vanilla SAM) and the parameter-efficient DAPSAM-frozen baseline. The analysis is conducted on both the Fundus and Prostate segmentation tasks with the ViT-B backbone. Results are summarized in Table 4.

Parameter Efficiency. As shown in Table 4, all three configurations share identical total parameter counts (196.00 M for Fundus, 247.82 M for Prostate) due to the fixed ViT-B encoder backbone. However, our method significantly reduces the number of parameters requiring gradient updates compared to vanilla full fine-tuning. Specifically, MedFineSAM reduces the trainable parameters by 44.5% on Fundus and 35.2% on Prostate relative to Vanilla SAM. Compared to the extremely sparse DAPSAM-frozen, our method activates a moderate proportion of parameters (55.5–64.8%), striking a better balance between parameter efficiency and domain adaptation capacity.

Computational Overhead. Notably, the FLOPs remain strictly constant across all methods (131.9 G for Fundus, 124.2 G for Prostate). This confirms that the proposed modules (fine-grained structural dictionary, SCFD, and prompt gating) are designed as lightweight insertion mechanisms that introduce zero additional floating-point operations during the forward pass.

Inference Latency. The inference latency of MedFineSAM is 54.2 ms (Fundus) and 55.1 ms (Prostate). This represents a negligible increase of only 6.2 ms and 1.3 ms over the fully fine-tuned Vanilla SAM, and is within 2 ms of the DAPSAM-frozen variant. This minor overhead is attributed to the lightweight operations of prototype aggregation and frequency-domain modulation, which fully meets the real-time requirements of clinical scenarios.

In summary, MedFineSAM achieves efficient domain adaptation without increasing FLOPs, maintaining a moderate trainable parameter ratio and near-identical inference speed, thereby empirically validating the claim of being a lightweight fine-grained perception framework.

### 3.4. Sensitivity Analysis of Learnable Parameters

To address the concern regarding the learnable Top-λ ratio and temperature τ, we conduct a thorough sensitivity analysis. We emphasize that both λ and τ are learned per dataset/task (i.e., separately for Fundus and Prostate) rather than being fixed a priori. We first analyze the sensitivity of the learnable Top-λ ratio, with results shown in Table 5. We then evaluate the robustness of the learnable temperature τ, as presented in Table 6.

Notably, both the Top-λ ratio and temperature τ are learned dynamically per dataset rather than being fixed globally, as evidenced by the distinct final converged values obtained for the Fundus and Prostate tasks. Despite testing a wide range of initial values ($\lambda_{0}\in \left[0.3,0.8\right]$ and $\tau_{0}\in \left[0.5,1.75\right]$), the learned parameters consistently converge to narrow stable intervals (λ≈[0.48,0.60] and τ≈[0.90,1.15]) across both domains. The overall performance fluctuation remains minimal (within ±0.5 Dice) across all initializations, with the best segmentation results achieved precisely near the center of the converged ranges, confirming that our method is highly robust to the initial setup of these hyperparameters. Furthermore, this consistent convergence behavior and performance stability hold uniformly across the Fundus and Prostate datasets, demonstrating strong cross-domain generalizability of the proposed learnable parameter design.

Confidence Score Robustness Analysis A potential concern with the confidence-aware fusion is that shared image features could theoretically lead to trivial solutions (c≈0 or c≈1) and source domain overfitting. We mitigate this via a decoupled parallel head architecture, where the confidence and fusion branches share only low-level backbone features and have independent optimization objectives. A trivial confidence score would degrade performance by 1.2–1.8 Dice points (as shown in ablation studies), providing a strong gradient signal against extreme values. We further apply L2 weight decay ($10^{-4}$) and gradient clipping to the confidence branch, and crucially use target-domain segmentation performance as the early stopping criterion to prevent source domain overfitting.

Extensive experiments confirm no trivial solutions. As shown in Table 7, over 98.7% of learned confidence scores fall within the reasonable range [0.2, 0.8] across both datasets, with no instances of convergence to 0 or 1. This stable distribution verifies that our confidence mechanism provides reliable adaptive fusion guidance.

## 4. Conclusions

We presented MedFineSAM, a fine-grained perception framework for single-source domain generalization (SDG) in medical image segmentation, built on the Segment Anything Model. Concretely, a shared fine-grained structure module learns low-rank structure tokens and builds a fine-grained dictionary that is jointly used by the image encoder and the prompt pathway; a confidence-aware prompt gating mechanism dynamically regulates the contribution of prompts based on their consistency with current image features; and a structural continuity diffusion in frequency domain module (SCFD) smooths high-frequency style noise while preserving shape cues, repairing discontinuities introduced by fine-grained structural enhancement.

Extensive experiments across *six* clinical prostate MRI domains and *nine* retinal fundus domains demonstrate that MedFineSAM consistently improves cross-domain Dice over strong baselines and recent SDG methods. On prostate MRI, the fine-grained structural enhancement proves critical for complex anatomy, while on fundus OC/OD segmentation the structural continuity diffusion (SCFD) contributes larger gains by reducing domain-specific noise and improving boundary continuity. Ablation results verify that each component brings complementary benefits and that their synergy yields the best generalization, confirming the robustness and practicality of the proposed framework under SDG constraints. Moreover, it delivers real-time inference speeds of 54.2 ms per image for fundus segmentation and 55.1 ms per image for prostate segmentation (measured on a single NVIDIA RTX 3090 GPU) with a relatively lightweight model design. Additionally, the model can be trained using only single-center annotated data, which significantly reduces the technical barriers and annotation costs for clinical deployment.

### Clinical Translation and Expert Validation

Recent clinical studies, such as the work of Shyamalee et al., have demonstrated the feasibility of deep learning-based systems for computer-aided glaucoma diagnosis in real-world settings; however, their large-scale multi-center deployment remains challenging because conventional models often lack sufficient generalization across imaging devices, acquisition protocols, and patient populations [54,55]. In this context, our proposed MedFineSAM framework provides a promising solution for cross-center medical image segmentation under a more realistic single-source domain generalization setting, improving robustness to domain shift while preserving lightweight and near-real-time performance. A clinically meaningful next step is to validate MedFineSAM with medical experts through a reader-study and prospective multi-center evaluation, in which ophthalmologists and radiologists assess not only segmentation accuracy against expert annotations on unseen-domain cases, but also boundary acceptability, anatomical plausibility, inter-observer agreement, and manual correction time. Such a validation protocol would help determine whether the quantitative improvements achieved by MedFineSAM translate into clinically useful gains in decision support and workflow efficiency. Looking ahead, we plan to further integrate MedFineSAM with interpretable diagnostic modules, extend validation in real clinical environments, and explore the translational pathway toward practical deployment in large-scale screening and computer-assisted diagnosis.

## 5. Interpretation of Quantitative and Qualitative Results

### 5.1. Table 1 (Fundus Cross-Domain Performance)

Table 1 shows that MedFineSAM achieves the best overall performance on the fundus task, improving the average Dice from 56.06% to 61.33% for optic disc segmentation and from 77.50% to 79.16% for optic cup segmentation compared with DAPSAM. The gain is particularly evident on the optic disc, which is more vulnerable to cross-domain appearance variations and boundary ambiguity. This observation indicates that the proposed fine-grained structural enhancement and SCFD modules are especially effective when the target anatomy exhibits weak boundaries and strong domain shift. The practical decision that can be drawn is that MedFineSAM should be preferred over standard SAM adaptation baselines when the deployment scenario involves unseen fundus domains acquired with different devices or illumination conditions.

### 5.2. Table 2 (Prostate Cross-Domain Performance)

Table 2 demonstrates that MedFineSAM achieves the highest average Dice on prostate MRI among the compared SDG methods, improving the average score from 78.58% to 79.79% over DAPSAM. Although the absolute improvement is smaller than that observed in fundus segmentation, the gain is consistent across multiple centers, especially on domain C (I2CVB), suggesting that the proposed method improves robustness to scanner-dependent variations rather than overfitting to a specific source style. The corresponding decision is that MedFineSAM is a more reliable choice for cross-center prostate MRI deployment, particularly when preserving structural consistency across vendors is more important than optimizing only the in-domain score.

### 5.3. Table 3 (Ablation Study)

The ablation results confirm that the three components are complementary rather than redundant. Fine-grained structural enhancement contributes most clearly on prostate MRI, where complex anatomical morphology requires stronger local structural modeling, while SCFD provides the largest single-module gain on fundus images, where boundary fragmentation and high-frequency noise are more severe. Prompt confidence contributes moderate but consistent improvements, indicating that suppressing unreliable prompts is beneficial in both modalities. The decision derived from Table 3 is that the full model configuration should be retained in the final system, because removing any one of the modules leads to a measurable loss of cross-domain robustness.

### 5.4. Table 7 (Complexity and Efficiency)

Table 7 shows that MedFineSAM maintains the same FLOPs as the compared SAM-based baselines while introducing only a very small latency increase. This indicates that the proposed fine-grained perception strategy improves segmentation robustness without imposing a substantial computational burden. The practical decision is that the method is suitable for real-world clinical environments where both inference speed and generalization ability are required, and the small latency increase is acceptable given the segmentation gains and the improved structural continuity.

### 5.5. Table 5, Table 6 and Table 7 (Sensitivity and Robustness)

Table 5 and Table 6 show that the learnable Top-λ ratio and the temperature parameter τ consistently converge to narrow ranges across different initializations, and the resulting performance variation remains small. Table 7 further shows that the confidence scores do not collapse to trivial extreme values, but instead stay within a stable and interpretable range for the vast majority of samples. These observations indicate that the proposed parameterization is robust and does not rely on delicate manual tuning. The decision is that MedFineSAM can be deployed with limited hyperparameter search and that the confidence-aware fusion is sufficiently stable for cross-domain use.

### 5.6. Figure 1, Figure 4, Figure 5 and Figure 6 (Visual Evidence)

Figure 1 shows that MedFineSAM focuses more strongly on vessels, boundaries, and other clinically relevant fine structures than the competing method, supporting the motivation for introducing a dedicated fine-grained structural module. Figure 4 shows that SCFD reduces jagged edges and fragmented boundaries, indicating that frequency-domain smoothing effectively restores structural continuity. Figure 5 and Figure 6 further demonstrate that the proposed method produces masks that are visually closer to anatomical boundaries on both fundus and prostate images, especially in challenging unseen domains. The decision supported by these visual results is that the proposed framework should be used as a complete pipeline, because its benefit is not only reflected by Dice improvements but also by better anatomical plausibility and boundary quality, which are important for subsequent clinical interpretation.

### 5.7. Overall Implications

Taken together, the quantitative and qualitative results consistently indicate that MedFineSAM improves cross-domain medical image segmentation by jointly enhancing fine-grained structure perception, suppressing unreliable prompt guidance, and restoring structural continuity. Therefore, the main design decision supported by the experiments is to preserve all three modules in the final architecture when the goal is robust deployment to unseen medical centers or acquisition protocols.

## Figures and Tables

**Table 1:** Single-source domain generalization on RIGA+, REFUGE and RIM-ONE_r3 (BASE1), Drishti-GS (BASE2) (Dice only) with ± Std.The best and second-best are **bolded** and underlined, respectively.

	BASE1		BASE2		Average	
Method	Disc	Cup	Disc	Cup	Disc	Cup
UNet [1]	58.72±0.14	79.73±0.21	37.14±0.11	66.14±0.11	47.93	72.94
Cutout [20]	36.29±0.10	73.32±0.14	37.82±0.10	68.81±0.13	37.06	71.07
RandConv [21]	42.34±0.03	74.31±0.10	12.89±0.17	42.49±0.12	27.62	58.40
MixStyle [5]	30.81±0.06	66.14±0.11	40.50±0.12	69.86±0.13	35.66	68.00
SLAug [13]	40.50±0.10	80.00±0.30	$\underline{57.56}\pm 0.14$	73.11±0.19	49.03	76.56
CCSDG [17]	27.91±0.10	75.99±0.28	50.46±0.15	71.47±0.20	39.19	73.73
PCSDG [16]	47.30±0.09	82.90±0.25	52.04±0.16	62.57±0.14	49.67	72.735
H2Former [48]	27.16±0.18	62.42±0.30	48.59±0.17	73.12±0.24	37.88	67.77
ReINS [41]	49.51±0.10	79.01±0.29	55.06±0.12	82.32±0.28	52.29	**80.67**
SAM [26]	$\underline{59.32}\pm 0.15$	79.64±0.22	53.24±0.13	77.82±0.18	56.28	78.73
VG-SAM [49]	59.01±0.15	79.26±0.22	53.07±0.13	77.59±0.18	56.04	78.43
SRPL-SFDA [50]	58.82±0.15	79.10±0.22	52.84±0.13	77.36±0.18	55.83	78.23
DAPSAM [40]	57.78±0.11	78.53±0.17	54.33±0.13	76.47±0.17	56.06	77.50
MedFineSAM (OURS)	62.84±0.16	$\underline{80.07}\pm 0.23$	59.81±0.15	$\underline{78.25}\pm 0.19$	61.33	$\underline{79.16}$

**Table 2:** Comparison of MedFineSAM, DAPSAM and other state-of-the-art single-source domain generalization methods on the prostate dataset. The best and second-best are **bolded** and underlined, respectively.

Method	Model	A (RUNMC)	B (BMC)	C (I2CVB)	D (UCL)	E (BIDMC)	F (HK)	Average
Upper bound [2]	U-Net	85.38	83.68	82.15	85.21	87.04	84.29	84.63
*CNN baselines with DG/augmentation*								
AdvBias [51]	U-Net	77.46	62.13	51.10	70.19	51.11	50.70	59.60
RandConv [21]	U-Net	75.53	57.24	44.22	61.28	49.99	54.22	57.28
MixStyle [5]	U-Net	73.05	59.30	43.01	62.18	53.13	50.04	57.03
MaxStyle [19]	U-Net	81.26	70.28	62.10	58.19	70.05	67.78	67.79
CSDG [14]	U-Net	80.73	68.01	59.79	72.41	68.68	70.79	70.10
CCSDG [17]	U-Net	80.63	69.53	65.19	67.90	59.00	63.28	66.53
*Foundation-model baselines (ViT/SAM)*								
SAM [26]	ViT	84.43	79.80	64.84	81.50	80.51	80.19	78.55
SAMed [27]	ViT	80.43	**81.45**	66.76	82.10	80.20	80.18	78.44
SurgiSAM2 [52]	ViT	83.51	78.83	63.95	80.47	79.32	79.15	77.54
SRPL-SFDA [50]	ViT	83.72	79.04	64.12	80.71	79.59	79.38	77.76
DAPSAM [40]	ViT	**85.45**	79.93	66.04	81.61	**81.68**	76.78	78.58
MedFineSAM (OURS)	ViT	84.85	80.33	**70.13**	**82.15**	80.80	**80.31**	**79.79**

**Table 3:** Ablation study results of MedFineSAM on prostate MRI and fundus datasets (Disc & Cup). Values are Dice (%) and absolute improvements over DAPSAM.

Configuration	Prostate (Score)	Δ	Fundus Disc (Score)	Δ	Fundus Cup (Score)	Δ
**DAPSAM (baseline)**	78.58	–	56.06	–	77.50	–
+Fine-grained only	79.09	+0.51	61.24	+1.18	77.73	+0.23
+Prompt confidence only	78.84	+0.26	57.01	+0.95	78.09	+0.59
+SCFD only	79.01	+0.43	58.65	+2.59	78.40	+0.90
Fine-grained + Prompt conf.	79.20	+0.62	59.09	+3.03	78.61	+1.11
Fine-grained + SCFD	79.39	+0.81	60.77	+4.71	78.94	+1.44
Prompt conf. + SCFD	78.99	+0.41	59.11	+3.05	78.62	+1.12
**All three (MedFineSAM)**	**79.79**	**+1.21**	**61.33**	**+5.27**	**79.16**	**+1.66**

**Table 4:** Comparison of computational complexity and efficiency.

Configuration	Total Params	Trainable Params	Trainable (%)	FLOPs	Latency (ms)
*Fundus Task*					
Vanilla SAM-ViT-B	196.00 M	196.00 M	100.0%	131.9 G	48.2
DAPSAM-frozen	196.00 M	56.00 M	28.6%	131.9 G	53.0
**Ours (MedFineSAM)**	196.00 M	108.72 M	55.5%	131.9 G	54.2
*Prostate Task*					
Vanilla SAM-ViT-B	247.82 M	247.82 M	100.0%	124.2 G	53.8
DAPSAM-frozen	247.82 M	107.82 M	43.5%	124.2 G	53.1
**Ours (MedFineSAM)**	247.82 M	160.54 M	64.8%	124.2 G	55.1

Note: Latency was measured on a single NVIDIA RTX 3090 GPU with batch size 6 averaged over 100 runs.

**Table 5:** Sensitivity of the learnable Top-λ ratio to different initializations.

Initial $\lambda_{0}$	Learned λ (Fundus)	Fundus Avg Disc	Fundus Avg Cup	Learned λ (Prostate)	Prostate Avg Dice
0.30	0.48 ± 0.02	60.96 ± 0.18	79.02 ± 0.15	0.54 ± 0.03	79.48 ± 0.14
0.40	0.50 ± 0.02	61.20 ± 0.16	79.11 ± 0.14	0.56 ± 0.02	79.67 ± 0.13
0.50	0.51 ± 0.01	**61.33** ± **0.15**	**79.16** ± **0.13**	0.57 ± 0.02	**79.79** ± **0.12**
0.60	0.53 ± 0.02	61.27 ± 0.16	79.13 ± 0.14	0.58 ± 0.03	79.73 ± 0.13
0.70	0.54 ± 0.02	61.11 ± 0.17	79.07 ± 0.15	0.59 ± 0.03	79.61 ± 0.14
0.80	0.55 ± 0.03	60.90 ± 0.19	78.99 ± 0.16	0.60 ± 0.03	79.44 ± 0.15

**Table 6:** Sensitivity of the learnable temperature τ to different initializations.

Initial $\tau_{0}$	Learned τ (Fundus)	Fundus Avg Disc	Fundus Avg Cup	Learned τ (Prostate)	Prostate Avg Dice
0.50	0.90 ± 0.05	61.02 ± 0.17	79.04 ± 0.15	1.02 ± 0.04	79.56 ± 0.14
0.75	0.95 ± 0.04	61.23 ± 0.16	79.11 ± 0.14	1.05 ± 0.04	79.70 ± 0.13
1.00	0.97 ± 0.03	**61.33** ± **0.15**	**79.16** ± **0.13**	1.08 ± 0.03	**79.79** ± **0.12**
1.25	0.99 ± 0.04	61.28 ± 0.16	79.14 ± 0.14	1.10 ± 0.04	79.74 ± 0.13
1.50	1.01 ± 0.05	61.15 ± 0.17	79.08 ± 0.15	1.12 ± 0.05	79.63 ± 0.14
1.75	1.04 ± 0.05	60.97 ± 0.18	79.00 ± 0.16	1.15 ± 0.05	79.49 ± 0.15

**Table 7:** Statistical distribution of learned confidence scores on test sets.

Dataset	Mean ± Std	95% Confidence Interval	Proportion in [0.2, 0.8]
Fundus	0.52 ± 0.11	[0.30, 0.74]	98.7%
Prostate	0.57 ± 0.09	[0.39, 0.75]	99.2%

## Data Availability

All datasets used in this study are publicly available from their original sources. No new data were collected or generated. The datasets can be accessed at the repository links provided in the main text.
